# Decoy Receptor 3 Inhibits Monosodium Urate-Induced NLRP3 Inflammasome Activation *via* Reduction of Reactive Oxygen Species Production and Lysosomal Rupture

**DOI:** 10.3389/fimmu.2021.638676

**Published:** 2021-03-03

**Authors:** Yi-Gen Pan, Ming-Ting Huang, Ponarulselvam Sekar, Duen-Yi Huang, Wan-Wan Lin, Shie-Liang Hsieh

**Affiliations:** ^1^Institute of Immunology, College of Medicine, National Taiwan University, Taipei, Taiwan; ^2^Genomics Research Center, Academia Sinica, Taipei, Taiwan; ^3^Department of Pharmacology, College of Medicine, National Taiwan University, Taipei, Taiwan; ^4^Graduate Institute of Medical Sciences, Taipei Medical University, Taipei, Taiwan; ^5^Department and Graduate Institute of Pharmacology, National Defense Medical Center, Taipei, Taiwan; ^6^Institute of Clinical Medicine & Immunology Research Center, National Yang-Ming Chiao Tung University, Taipei, Taiwan; ^7^Department of Medical Research and Education, Taipei Veterans General Hospital, Taipei, Taiwan; ^8^Institute for Cancer Biology and Drug Discovery, Taipei Medical University, Taipei, Taiwan

**Keywords:** decoy receptor 3, NLRP3 inflammasome, lysosome, gout, reactive oxygen species, monosodium urate, cathepsin

## Abstract

Gout is a common inflammatory arthritis caused by the deposition of monosodium urate (MSU) crystals in the joints. This activates the macrophages into a proinflammatory state by inducing NLRP3-dependent interleukin-1β (IL-1β) secretion, resulting in neutrophil recruitment. Soluble decoy receptor 3 (DcR3) is an immune modulator and can exert biological functions *via* decoy and non-decoy actions. Previously, we showed that DcR3 suppresses lipopolysaccharides (LPS)- and virus-induced inflammatory responses in the macrophages and promotes the macrophages into the M2 phenotype. In this study, we clarified the actions of DcR3 and its non-decoy action motif *h*eparin sulfate proteoglycan (HSPG) *b*inding *d*omain (HBD) in the MSU crystal-induced NLRP3 inflammasome activation in the macrophages and in mice. In bone marrow-derived macrophages, THP-1 and U937 cells, we found that the MSU crystal-induced secretion of IL-1β and activation of NLRP3 were suppressed by both DcR3.Fc and HBD.Fc. The suppression of the MSU-induced NLRP3 inflammasome activation is accompanied by the inhibition of lysosomal rupture, mitochondrial production of the reactive oxygen species (ROS), expression of cathepsins, and activity of cathepsin B, without affecting the crystal uptake and the expression of NLRP3 or pro-IL-1β. In the air pouch mice model of gout, MSU induced less amounts of IL-1β and chemokines secretion, an increased M2/M1 macrophage ratio, and a reduction of neutrophil recruitment in DcR3-transgenic mice, which expresses DcR3 in myeloid cells. Similarly, the mice intravenously treated with DcR3.Fc or HBD.Fc displayed less inflammation response. These findings indicate that HBD of DcR3 can reduce MSU crystal-induced NLRP3 inflammasome activation *via* modulation of mitochondrial and lysosomal functions. Therefore, we, for the first time, demonstrate a new therapeutic potential of DcR3 for the treatment of gout.

## Introduction

Decoy receptor 3 (DcR3) is a soluble receptor belonging to the tumor necrosis factor receptor (TNFR) superfamily. The immunomodulatory functions of DcR3 can be divided into decoy and non-decoy functions (1). The decoy function is attributed to the neutralization ability of DcR3 to interact with three members of the tumor necrosis factor (TNF) superfamily: Fas Ligand (FasL), LIGHT, and TNF-like Ligand 1A (TL1A). The decoy action of DcR3 has been shown to promote the progression of tumors. It has been shown that malignant cells secrete high levels of DcR3 to block FasL-mediated cell death (2, 3). Other studies also showed that DcR3 can block the LIGHT–Herpes virus entry mediator (HVEM) interaction to attenuate alloantigen-induced interleukin-2 (IL-2) secretion of T cells (4) and promote angiogenesis by neutralizing TL1A (5). Moreover, transgenic mice overexpressing DcR3 showed less Th1-mediated response (3) and beta-amyloid-induced neuronal inflammation (6).

In addition to the neutralizing actions, endogenous DcR3 acts as an effector to modulate immune cell functions. DcR3.Fc-treated dendritic cells skew the T-cell differentiation into the Th2-predominant phenotype (7), and DcR3.Fc is able to drive the macrophage differentiation toward the M2 phenotype (8, 9) and promote the monocyte adhesion (10, 11). Moreover, DcR3.Fc can promote the differentiation of the monocytes/macrophages into osteoclasts (12) or the tumor-associated macrophages (13) and ameliorate experimental autoimmune encephalomyelitis (14). Moreover, recombinant DcR3.Fc can improve mice survival in experimental sepsis (15), promote locomotor functional recovery after spinal cord injury (16), and attenuate hepatic steatosis (17). Interestingly, previous studies indicate that the non-decoy effector functions of DcR3 are *via* its *h*eparin sulfate proteoglycan (HSPG) *b*inding *d*omain (HBD), which comprises a stretch of positive amino acid residues, to activate HSPGs, such as syndecan 2 and CD44v3 (11). Moreover, recombinant DcR3.Fc and HBD.Fc fusion proteins have similar abilities to reduce the influenza virus-induced lethality (18). Unlike DcR3.Fc, HBD.Fc only contains a stretch of positive charge of the amino acid (aa 256-261) of DcR3 located outside the ligand binding region (11). Thus, the HBD.Fc-mediated effect is independent of the neutralizing actions of DcR3 or DcR3.Fc.

Gout is a common inflammatory arthritis characterized by recurrent, sudden, and severe attacks of pain, redness, and tenderness at the joints. Gout is caused by the deposition of monosodium urate (MSU) crystals, which are ingested by the resident macrophages *via* phagocytosis in the joints, thereby leading to local inflammatory responses in the joint cavities, and the surrounding connective tissues (19). Internalized MSU crystals cause lysosomal destabilization and rupture, thereby releasing lysosomal proteases and cathepsins (20). The cytosolic cathepsin then directly interacts with NLRP3 for inflammasome activation and the production of IL-1β (21, 22). In addition, the internalized MSU crystals trigger the production of reactive oxygen species (ROS), release of ATP, and the activation of purinergic signaling (23, 24), which are critical for the assembly of NLRP3 inflammasome. Furthermore, crystals can modulate oxidative stress by activating the NADPH oxidase upon particle phagocytosis (25), impair mitochondrial transmembrane potential (26, 27), increase mitochondrial ROS generated through the fatty acid oxidation (28), and induce cathepsin B release (29). Based on these observations, IL-1β blockers are considered promising candidates for the therapy of gout (30, 31). Our previous reports showed that DcR3.Fc modulates the differentiation of the macrophages toward M2-like phenotype and suppresses the secretion of proinflammatory cytokines such as tumor necrosis factor-alpha (TNF-α), interleukin 6 (IL-6), and interferon-alpha (IFN-α) after viral infections and stimulation of the lipopolysaccharides (LPS) (8, 9). However, it remains unclear whether DcR3.Fc and HBD.Fc are effective to suppress the MSU crystal-induced NLRP3 inflammasome activation.

In addition, macrophage colony-stimulating factor (M-CSF) and granulocyte-macrophage colony-stimulating factor (GM-CSF) can skew the differentiation of the monocytes into the macrophages of the homeostatic and resting condition (denoted as resting M–Mϕ) and the inflammatory stage (denoted as inflammatory GM-Mϕ), respectively (32). Our previous study demonstrated that dengue virus–induced NLRP3 activation and IL-1β secretion are more pronounced in GM-Mϕ than M-Mϕ (33). These observations suggest that GM-Mϕ and M-Mϕ might react differentially to crystals of the MSU and particulate antigens. Another common way to have the M1-like primary macrophages is to culture the bone marrow–derived macrophages (BMDM) containing the L929 conditional medium (denoted as resting L929-Mϕ) (34).

In this study, we investigated the secretion of the MSU-induced IL-1β and the activation of caspase-1 in LPS-primed mouse GM-Mϕ, M-Mϕ, L929-Mϕ and the human THP-1 monocytes-derived macrophages. Moreover, MSU-induced phagocytosis, lysosomal stability, expression of cysteine cathepsins, and mitochondrial production of the ROS were also compared between the hIgG- and DcR3.Fc-treated macrophages. We also compared the extents of the inflammation and the tissue damage between the wild-type (WT) and the DcR3-transgenic mice using the MSU-induced air pouch mouse model. The effects of the recombinant DcR3.Fc and HBD.Fc on the MSU-induced inflammation in the macrophages and mice were also examined to reveal the decoy or non-decoy role of DcR3.Fc in the MSU crystal-induced NLRP3 activation and inflammatory reactions.

## Methods

### Reagents

Mouse M-CSF, mouse GM-CSF, human caspase-1, and mouse IL-1β, IL-6, CCL2, CXCL2, and CXCL1 ELISA kits were purchased from R&D Systems (Minneapolis, MN, USA). Uric acid and human IgG1 (hIgG) were obtained from Sigma-Aldrich (St. Louis, MO, USA). Anti-Ly6G Ab was purchased from BD Pharmingen (San Jose, CA, USA), anti-F4/80 was purchased from Serotec (Oxford, UK), anti-arginase 1 (#93668) was purchased from Cell Signaling Technology (Danvers, MA, USA), and anti-CD206 (AF2535) was purchased from R&D (Minneapolis, MN, USA). Silica (MIN-U-SIL-5; average particle diameter 1.7 μm) was obtained from US Silica Co. (Katy, TX, USA). Imject Alum was obtained from Thermo Scientific (Waltham, MA, USA).

### DcR3.Fc and HBD.Fc Fusion Proteins

DcR3.Fc and HBD.Fc fusion proteins were produced by baculovirus and the FreeStyle Expression system separately as we previously described (11, 18). Sf21 cells were infected by baculovirus containing DcR3.Fc expressing vector and the supernatant was collected at day 7. HBD.Fc fusion proteins were extracted from the supernatant of 293F cells 2 days after plasmid transfection. Both proteins were purified by protein A-Sepharose beads (Amersham Biosciences) and diluted with 0.1 M glycine buffer (pH 3.0). For all experiments, hIgG was used as a control for DcR3.Fc or HBD.Fc.

### DcR3-Transgenic Mice and Macrophage Cultures

DcR3-transgenic (tg) mice with DcR3 expression in myeloid cells were generated as previously described (13). Experiments with mice were conducted in accordance with the regulation of the institute after receiving the approval from the Ethics Committee of the National Taiwan University College of Medicine (No. 20180091). The bone marrow of B6 mice were collected and cultured in 10 cm dishes. Each dish contained 5 × 10^6^ cells in 10 ml high glucose Dulbecco's Modified Eagle Medium (DMEM) supplemented with 10% fetal bovine serum (FBS), 2 mM L-glutamine, 3.7 g/l sodium bicarbonate (NaHCO_3_), 100 U/ml penicillin, 100 μg/ml streptomycin, and 10 ng/ml recombinant mouse GM-CSF or M-CSF. Fresh medium with GM-CSF or M-CSF was added into each dish at day 3. BMDMs obtained after culturing for 6 days in mediums containing M-CSF or GM-CSF were referred to GM-Mϕ or M-Mϕ. In some experiments, bone marrow cells were cultured in complete DMEM using 10% conditional media of L929 cells to replace the GM-CSF and M-CSF. L929 medium has been demonstrated to promote the growth and differentiation of the BMDMs *in vitro* as the action of M-CSF (34). To understand the effect of DcR3, human IgG1, DcR3.Fc, or HBD.Fc (each at 1 or 3 μg/ml) was added in the medium at day 0 and day 3. THP-1 and U937 cells were cultured in RPMI 1640 medium containing 3 nM phorbol-12-myristate-13-acetate (PMA) with or without human IgG, DcR3.Fc, or HBD.Fc (1 or 3 μg/ml). After 24 h, cells were washed with phosphate-buffered saline (PBS) and used for experiments.

### Preparation of the MSU

MSU crystals were prepared by a modification protocol previously described (35). Uric acid was resolved in pre-warmed 30 mM sodium hydroxide solution. The solution was sterilized by passing through a 0.22 μm filter, and the dissolved uric acid was recrystallized by adjusting the pH value of the solution to 7.0 and cooling to room temperatures. The MSU crystal was washed with 75% ethanol and suspended in PBS for experiments.

### Cytokines and Caspase-1 Activity Assays

GM-CSF-derived and M-CSF–derived BMDMs were collected and 1 × 10^5^ cells were seeded in each well of 96-well plates. These cells were primed with 100 ng/ml LPS. After 4 h, LPS-primed cells were treated with MSU, silica, alum (150 or 300 μg/ml), or ATP (3 mM). The supernatant was collected 1 h after the treatment with ATP and 4 or 6 h after the treatment with crystals. For THP-1- or U937-derived macrophages, cells were primed with LPS (100 ng/ml) for 4 h followed by the treatment with the MSU (150 or 300 μg/ml) for 4 h. The concentrations of IL-1β and active caspase-1 in the culture medium were determined by using ELISA kits in accordance with the instructions of the manufacturer.

### Phagocytosis, Lysosomes, and Mitochondrial ROS

Phagocytosis of the MSU crystals was measured by flow cytometry. 1 × 10^6^ GM-Mϕ or M-Mϕ was seeded in 12-well plates and then was incubated with 300 μg/ml MSU crystals. Cells were washed extensively three times with cold PBS and harvested at 15 and 60 min after the treatment with MSU. The percentage of cells that phagocytized MSU crystals (300 μg/ml) was calculated from elevating side-scatter populations among MSU-treated cells compared to non-treated cells.

For evaluation of lysosomal rupture, 5 × 10^5^ M-Mϕ and GM-Mϕ were incubated for 15 min with acridine orange (AO, 1 μg/ml). Then, cells were washed with PBS and treated with the MSU (300 μg/ml) for 2 h. Cells were harvested and the fluorescence of AO in low pH organelle was measured by the R-PE (Phycoerythrin) channel. We also used LysoTracker Red DND-99 (5 μM) to measure lysosomal mass in L929-Mϕ as previously described (36). For evaluation of mitochondrial ROS, M-Mϕ, GM-Mϕ L929-Mϕ and THP-1 cells were incubated with MitoSOX Red (5 μM). Fluorescence signals were detected using flow cytometry (FACS Calibur system Franklin Lakes, NJ, USA).

### The Air Pouch Model

According to previous reports (37), the air pouch model was used for analyzing the role DcR3 on MSU-mediated inflammation *in vivo*. Briefly, WT or DcR3-tg B6 mice were anesthetized with 2% tribromoethanol (Avertin) and injected with 3 ml sterile air subcutaneously (s.c.) to form an air pouch on the back. The pre-existent air pouch was maintained and enlarged by 2 ml sterile air injection at day 3. A synovial-like epithelium was present in the air pouch at day 7. After 6 or 24 h, the infiltrated cells in the air pouch membrane were collected by injection with 2 ml cold PBS. Then, the cells in the air pouch fluid (exudate) were stained with anit-F4/80-APC or anti-Ly6G-FITC antibody and analyzed by BD FACSVerse™ flow cytometer (Franklin Lakes, New Jersey, US). Some samples were checked live/dead by trypan blue staining through microscopy, and no samples that contained more than 1% dead cells were found. The levels of cytokines (IL-1β and IL-6) and chemokines (CXCL1, CXCL2, and CCL2) were measured by ELISA. In some experiments, to determine the effect of exogenous DcR3, we injected DcR3.Fc or HBD.Fc (30 or 90 μg) intravenously into WT mice 24 h before PBS (1 ml) or 3 mg MSU crystals (in 1 ml PBS) was given into the air pouch.

### The Measurement of Lysosomal Rupture by Confocal Reflection Microscopy

Engulfed MSU crystals and lysosomal rupture observation were done by confocal reflection microscopy and lysosome-sensitive fluorogenic substrate, DQ-ovalbumin by following previous descriptions (38). In brief, 1 × 10^5^ day 6 M-Mϕ and GM-Mϕ were seeded on coverslip over 2 h for adhesion. Adherent cells were treated with DQ-ovalbumin (10 μg/ml) and MSU (300 μg/ml) at 37°C. After 2 h incubation, cover slides were fixed by 4% paraformaldehyde (PFA)/PBS for 1 h at 4°C. Then, cell nuclei were stained with Hoechst. All the images were captured by Leica TCS SP5 AOBS confocal laser scanning microscope. The reflection signals for MSU crystals were captured by setting the detection channel directly over the wavelength of the chosen laser to allow 5–15% light to pass.

### Real-Time PCR

Cells were harvested, and total RNAs were extracted using TRIzol reagents (Invitrogen) and converted to cDNA using the Revert Aid First Strand cDNA Synthesis Kits (Thermo Fisher Scientific) according to the instructions of the manufacturer. SYBR Green real-time PCR was performed with Luminaris Color HiGreen qPCR master mix by PikoReal System (Thermo Fisher Scientific). Primers used for the amplification of specific genes are listed below. All mRNA levels of target genes were normalized with GAPDH and the fold change represented the expression of the genes of agent-treated cells compared to that of untreated cells.

**Table:** 

**Target genes**	**Sequence**
Human pro-IL-1β	Forward: 5′- GGA TAT GGA GCA ACA AGT GG-3′ Reverse: 5′- GAA GTC AGT TAT ATC CTG GC-3′
Human NLRP3	Forward: 5′- CCA AGA ATC CAC AGT GTA ACC−3′ Reverse: 5′- CTT CAC AGA ACA TCA TGA CCC−3′
Human Cathepsin B	Forward: 5′- GAT CTG CAT CCA CAC CAA TG−3′ Reverse: 5′- GGA GGG ATG GAG TAC GGT CT−3′
Human Cathepsin F	Forward: 5′- CCC TCC AAT GCC TAC TCG G−3′ Reverse: 5′- CCA GCT TCT GCT CGT TCT G−3′
Human Cathepsin K	Forward: 5′- GGC CAA CTC AAG AAG AAA−3′ Reverse: 5′- GTA CCC TCT GCA TTT AGC−3′
Human GAPDH	Forward: 5′- CCA TCA CTG CCA CCC AGA AGA C−3′ Reverse: 5′- GGC AGG TTT TTC TAG ACG GCA G−3′
Mouse pro-IL-1β	Forward: 5′- CGG CAC ACC CAC CCT G−3′ Reverse: 5′- AAA CCG CTT TTC CAT CTT CTT CT−3′
Mouse NLRP3	Forward: 5′- CGA GAC CTC TGG GAA AAA GCT−3′ Reverse: 5′- GCA TAC CAT AGA GGA ATG TGA TGT ACA−3′
Mouse Cathepsin B	Forward: 5′- TCC TTG ATC CTT CTT TCT TGC C−3′ Reverse: 5′- ACA GTG CCA CAC AGC TTC TTC−3′
Mouse Cathepsin C	Forward: 5′- CAA CTG CAC CTA CCC TGA TC−3′ Reverse: 5′- CTC GTC GTA GGC AGT ATC CA−3′
Mouse Cathepsin F	Forward: 5′- GCA ACT TCT CAG CAC AGA TGG CAA−3′ Reverse: 5′- GAA CTG CAT GCC GAA GGC GTT AAT−3′
Mouse Cathepsin H	Forward: 5′- TAC AAC AAG GGC ATC ATG GA−3′ Reverse: 5′- TTC TTG ACG AAT GCA ACA GC−3′
Mouse Cathepsin K	Forward: 5′- ATG TGA ACC ATG CAG TGT TGG TGG−3′ Reverse: 5′- ATG CCG CAG GCG TTG TTC TTA TTC−3′
Mouse Cathepsin L	Forward: 5′- ATC AAA CCT TTA GTG CAG AGT G−3′ Reverse: 5′- CTG TAT TCC CCG TTG TGT AGC−3′
Mouse Cathepsin O	Forward: 5′- TGG TGG CAG ATT CAC AGT ACC CAT−3′ Reverse: 5′- AGT GCT CTG GCC ATT TCA TCC TCT−3′
Mouse Cathepsin S	Forward: 5′- AAG CGG TGT CTA TGA CGA CCC−3′ Reverse: 5′- GAG TCC CAT AGC CAA CCA CAA G−3′
Mouse Cathepsin Z	Forward: 5′- TAT GCC AGC GTC ACC AGG AAC−3′ Reverse: 5′- CCT CTT GAT GTT GAT TCG GTC TGC−3′
Mouse GAPDH	Forward: 5′- GAC AAC TTT GGC ATT GTG G−3′ Reverse: 5′- ATG CAG GGA TGA TGT TCT G−3′

### Histologic Analysis

The skins of the air pouch area were collected at 6 h after the injection with the MSU and were fixed in 10% paraformaldehyde PBS solution for over 2 days. The fixed tissues were paraffin embedded and sliced into sections for further staining of the H&E or experiments of immunohistochemistry (IHC). The H&E staining was done by the Taiwan Mouse Clinic of National Comprehensive Mouse Phenotyping and Drug Testing Center (Taipei, Taiwan). For experiments of IHC, embedded tissue sections were heated at 65°C for 16 h. Then, the sections were dewaxed and sequentially rehydrated by xylene (J.T. Baker, 9490-03) and ethanol (Millipore, 107017). The slides were blocked by the streptavidin/biotin blocking kit (Vector Lab, SP-2002) and incubated with Abs against arginase 1 or CD206 for 16 h. Then, conjugated anti-rabbit IgG or conjugated anti-goat IgG were used as secondary Abs, and the signals were developed by DAB system (Dako, K3468).

### Statistical Analysis

The data were presented as mean ± SD from independent experiments. The significance of data comparisons was performed through the two-tailed student's *t*-test. Multiple-way comparisons were performed using one-way analysis of variance (ANOVA) and corrected through *post-hoc* Tukey test for multiple comparisons. Statistical analyses were performed using the GraphPad Prism. ^*^p < 0.05, ^**^p < 0.01, ^***^p < 0.001.

## Results

### The Suppression of the Secretion of IL-1β in the MSU-Stimulated Macrophages by DcR3.Fc and HBD.Fc

**Figure 1:**
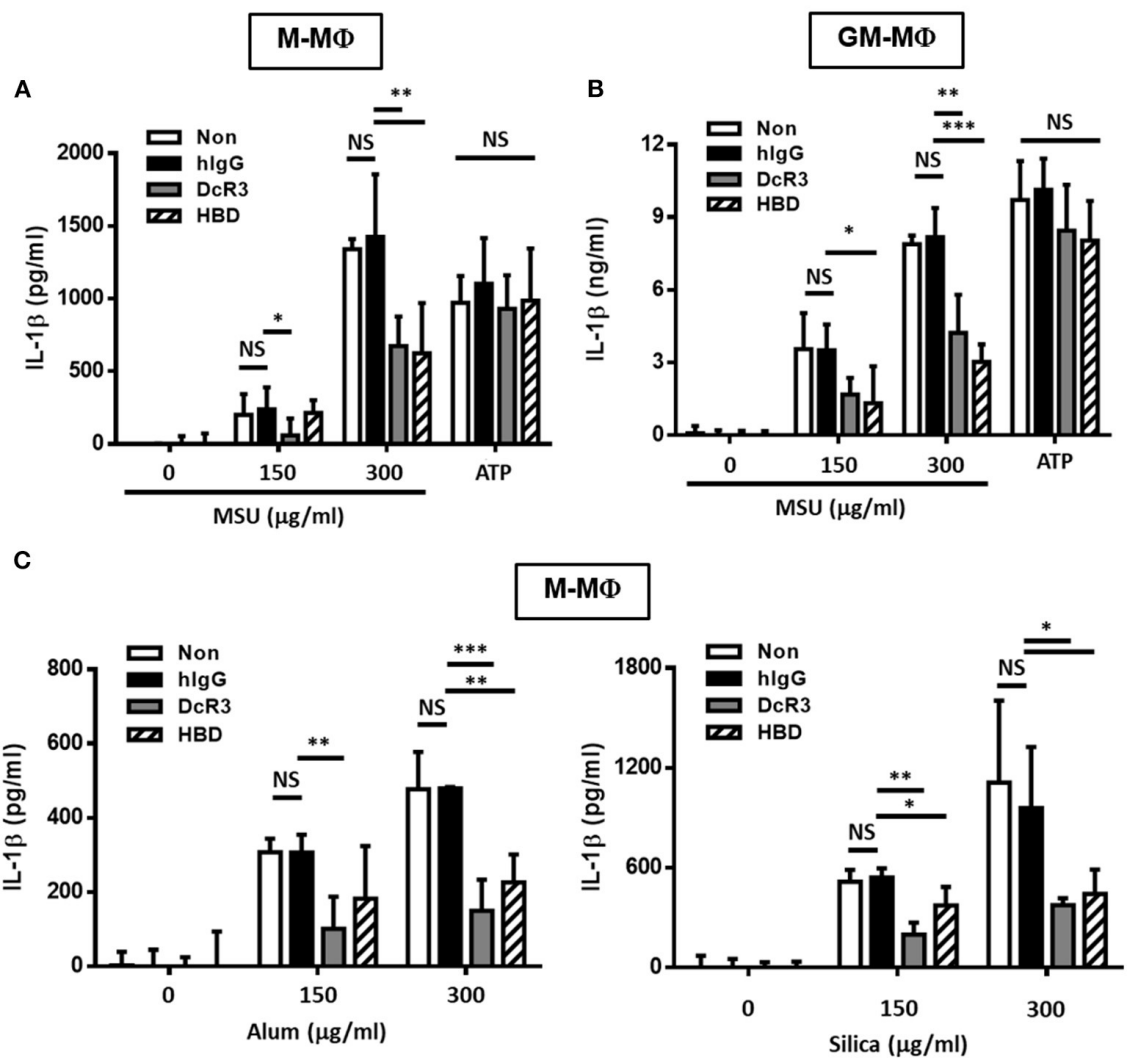
DcR3.Fc- or HBD.Fc-treated M-Mϕ and GM-Mϕ secreted low amount of interleukin-1β (IL-1β) under particle stimulation. Six-day-cultured M-Mϕ **(A,C)** and GM-Mϕ **(B)** were derived from wild-type (WT) B6 and cocultured with hIgG (3 μg/ml), DcR3.Fc (3 μg/ml), or HBD.Fc (3 μg/ml). Cells were primed with lipopolysaccharides (LPS; 100 ng/ml) for 4 h and treated with monosodium urate (MSU) crystal, alum, or silica (150 or 300 μg/ml) for 6 h or ATP (3 mM) for 1 h. The concentration of IL-1β was measured by ELISA. All data shown were mean ± SD from three independent experiments. The statistical significance was determined by one-way ANOVA. *p < 0.05, **p < 0.01, and ***p < 0.001 were obtained by comparing the DcR3.Fc- or HBD.Fc-treated group to the hIgG-group. “NS” means no statistical significance.

To understand the immunomodulatory effects of DcR3 on gout, we compared the effects of DcR3.Fc and HBD.Fc on MSU-induced IL-1β secretion in LPS-primed M-Mϕ and GM-Mϕ. We found that MSU induced higher levels of IL-1β in LPS-primed GM-Mϕ than M-Mϕ. The secretion of IL-1β was suppressed by DcR3.Fc and HBD.Fc (3 μg/ml each) in M-Mϕ incubated with MSU (300 μg/ml) but not with ATP (3 mM) (Figures 1A,B). Similar observations were shown in PMA-treated THP-1 and U937 cells (Supplementary Figure 1). In addition, we found that IL-1β levels were much higher in GM-Mϕ and THP-1 than M-Mϕ and U937. Similarly, inhibitory effects of DcR3.Fc and HBD.Fc were observed in M-Mϕ treated with other particulate antigens, such as silica and alum (Figure 1C). These observations suggest that DcR3.Fc and HBD.Fc can modulate crystal-induced but not ATP-induced, inflammasome activation. Given that DcR3.Fc and HBD.Fc have similar inhibitory effects on crystal-mediated IL-1β secretion, we suggest this phenomenon of DcR3 is through its non-decoy action.

### DcR3.Fc and HBD.Fc Suppress NLRP3 Inflammasome Activation but Not NLRP3 or the Induction of Pro-IL-1β in LPS-Stimulated Macrophages

**Figure 2:**
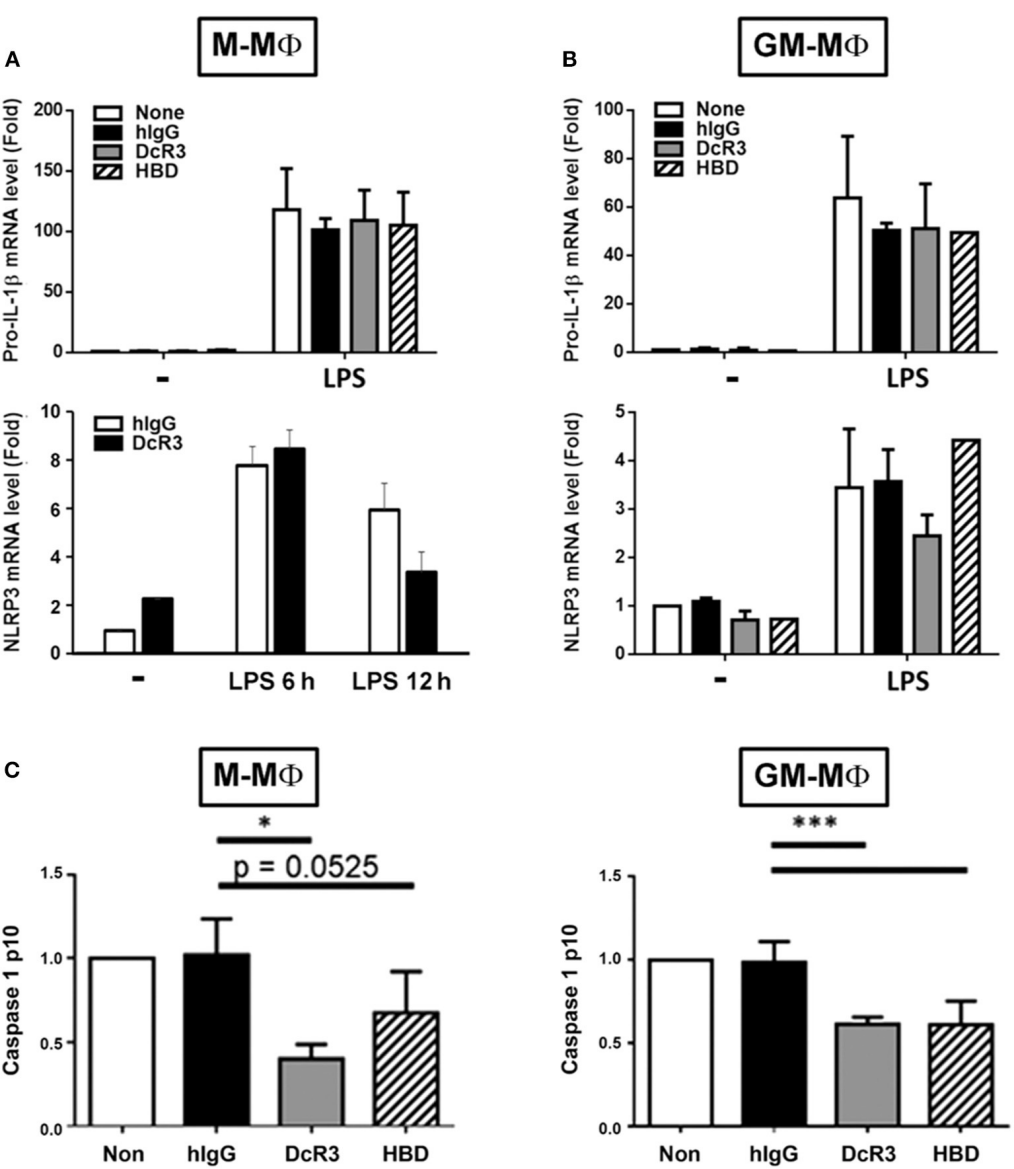
DcR3 suppressed MSU-induced caspase-1 activation but did not affect the expression of NLRP3 or pro-IL-1β in LPS-treated macrophages. M-Mϕ **(A,C)** and GM-Mϕ **(B,C)** were treated with hIgG, DcR3.Fc, or HBD.Fc (3 μg/ml for each) for 6 days during differentiation stage in M-Mϕ and GM-Mϕ. Cells were treated with LPS (100 ng/ml) for 4 h, and total RNA was extracted. The mRNA levels of pro-IL-1β and NLRP3 were measured by real-time PCR **(A,B)**. In some experiments, after LPS priming, cells were treated with MSU (300 μg/ml) for 3 h. The supernatants were harvested for caspase 1 p10 ELISA analysis **(C)**. Data indicated mean ± SD of three independent experiments. The statistical significance was determined by one-way ANOVA. *p < 0.05 and ***p < 0.001 were obtained by comparing DcR3.Fc- or HBD.Fc-treated group to hIgG-group.

Previous studies demonstrated that MSU induced NLRP3 inflammasome activation *via* triggering both signal 1 (pro-IL-1β and NLRP3 induction) and signal 2 (NLRP3 assembling and pro-caspase 1 cleavage) (39). Thus, we asked whether DcR3.Fc and. HBD.Fc can modulate signal-1 and/or signal-2 events. First, we analyzed the gene expression of pro-IL-1β and NLRP3 in BMDMs after treatment with LPS. We found that the levels of pro-IL-1β and NLRP3 mRNA were increased in LPS-primed cells, but DcR3.Fc and HBD.Fc did not affect LPS-induced expression of the pro-IL-1β and NLRP3 genes in M-Mϕ (Figure 2A) and GM-Mϕ (Figure 2B). We further asked whether DcF3.Fc or HBD.Fc can modulate the assembling of NLRP3 and pro-caspase 1 cleavage by determining active caspase-1 p10 in the culture medium. We found that DcR3.Fc and/or HBD.Fc suppressed the release of caspase-1 p10 in MSU-stimulated M-Mϕ and GM-Mϕ (Figure 2C). Thus, we conclude that the inhibitory effects of DcR3.Fc and HBD.Fc on the production of IL-1β are *via* the suppression of signal 2 in MSU-treated macrophages.

### The Engulfment of MSU Was Not Changed in DcR3/HBD-Treated Macrophages

**Figure 3:**
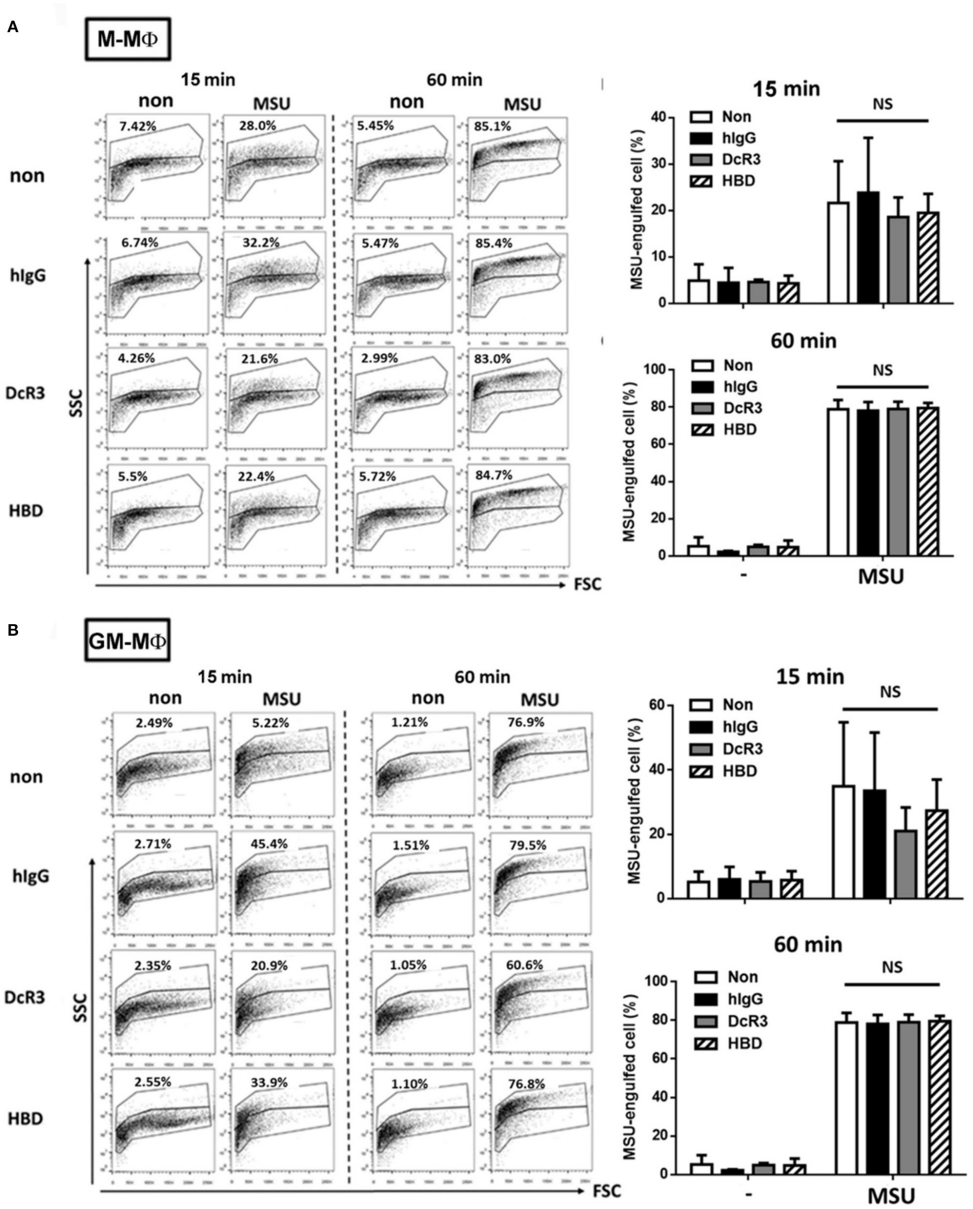
DcR3 did not suppress MSU crystal phagocytosis by the macrophages. M-Mϕ **(A)** and GM-Mϕ **(B)** were treated with hIgG, DcR3.Fc, or HBD.Fc (3 μg/ml for each) for 6 days. Cells were then treated with 300 μg/ml MSU for indicated times. The phagocytosis of MSU crystals was analyzed by the increase of SSC relative to control cells. The percentages of MSU-engulfed cells were calculated from markers that defined non-overlapping side-scatter populations between control and MSU-treated cells and were indicated in each dot plot. All data shown were mean ± SD from three independent experiments. The statistical significance was determined by one-way ANOVA. “NS” indicates no statistical significance by comparing DcR3.Fc- or HBD.Fc-treated group to hIgG-group.

It was MSU-induced but not ATP-induced IL-1β secretion was inhibited by DcR3.Fc and HBD.Fc. This observation indicated that the ion flux– and purinergic signaling–mediated inflammasome activation are unlikely targets of DcR3.Fc and HBD.Fc. Herein, we checked whether DcR3.Fc and HBD.Fc suppressed the secretion of IL-1β *via* the inhibition of the phagocytosis of MSU crystals by the macrophages. To address this question, DcR3.Fc- or HBD.Fc-treated M-Mϕ and GM-Mϕ were incubated with MSU crystals at 37°C, then examined through the engulfment of MSU using flow cytometry. The MSU crystal-engulfing cells were characterized by increasing the side scatter (SSC) values. As a result, DcR3.Fc and HBD.Fc did not affect the engulfment of MSU in M-Mϕ (Figure 3A) and GM-Mϕ (Figure 3B), suggesting the reduced secretion of IL-1β by DcR3.Fc and HBD.Fc is not due to the impairment of the uptake of MSU.

### The Suppression of MSU-Induced Lysosomal Rupture by DcR3.Fc and HBD.Fc

**Figure 4:**
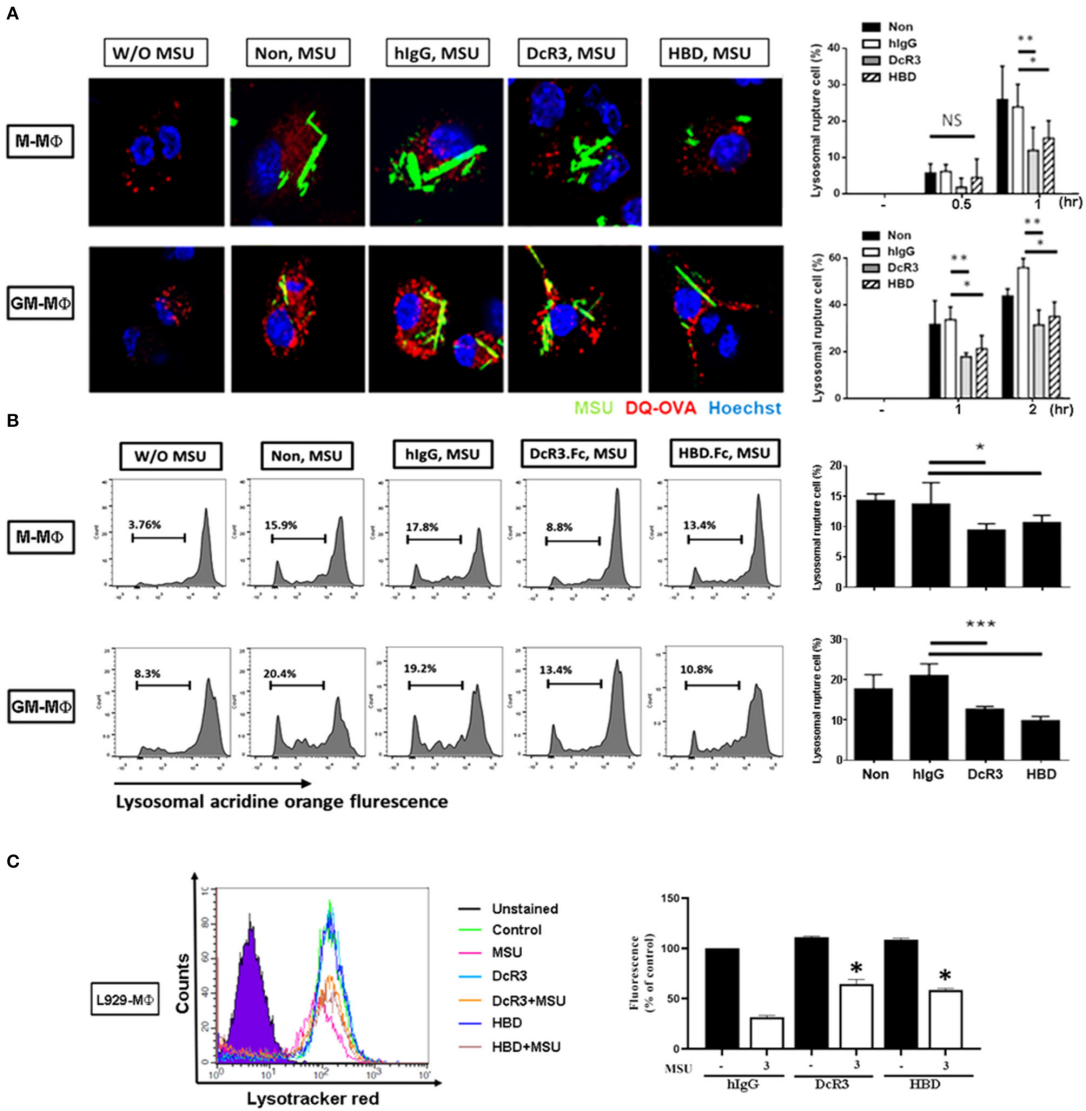
DcR3 reduced MSU-induced lysosomal rupture in the macrophages. **(A)** M-Mϕ and GM-Mϕ were incubated with hIgG, DcR3.Fc or HBD.Fc (3 μg/ml for each) for 6 days and were treated with DQ-OVA (10 μg/ml, red) and MSU (300 μg/ml, green) for 0.5, 1, or 2 h. Cells were stained with Hoechst dye (blue). The images were obtained by confocal microscopy under 250X magnification. **(B)** M-Mϕ and GM-Mϕ were stained with acridine orange (AO, 1 μg/ml) and then treated with MSU (300 μg/ml) for 2 h. **(C)** After treatment with MSU (300 μg/ml) for 2 h, L929-Mϕ was stained with Lysotracker™ Red DND-99. Fluorescence signals were detected using flow cytometry (FACS Calibur system Franklin Lakes, NJ, USA) and represented as percentages of control group. All the data were represented as one of three independent experiments. All data shown were mean ± SD of three independent experiments. The statistical significance was determined by one-way ANOVA. *p < 0.05, **p < 0.01, and ***p < 0.001 were obtained by comparing DcR3.Fc- or HBD.Fc-treated group to hIgG-group. “NS” indicates no statistical significance.

After the engulfment, MSU would fuse with lysosomes and cause lysosomal destabilization and rupture, thereby leading to inflammasome activation. To understand whether lysosomal integrity is affected by DcR3.Fc or HBD.Fc upon the engulfment of MSU, we used confocal reflection microscopy to examine proteolytic degradation of DQ-ovalbumin (indicated using red) after the engulfment of MSU (indicated using green) in macrophages. While normal lysosomes are small with strong signals, ruptured lysosomes are large and swollen with weaker signals. To quantify the extent of the lysosomal rupture, the number of cells containing ruptured lysosomes was counted under an inverted fluorescence microscope. We found that the MSU-induced lysosomal rupture was attenuated in DcR3.Fc- and HBD.Fc-treated M-Mϕ and GM-Mϕ (Figure 4A). Similar findings were observed in M-Mϕ and GM-Mϕ stained with AO which is present in acidic vesicles and used to trace the fate of lysosomes (Figure 4B). To confirm these observations, the macrophages cultured under L929 condition medium (L929-Mϕ) were incubated with Lysotracker™ Red to detect the lysosomal destabilization after treatment with MSU. We found that the lysosomal destabilization was inhibited by DcR3.Fc and HBD.Fc in L929-Mϕ (Figure 4C). Thus, we conclude that DcR3.Fc and HBD.Fc can modulate lysosomal stability to attenuate the activation of NLRP3 caused by the MSU crystals.

### The Expression of Cathepsin F and K and the Activation of Cathepsin B Were Suppressed by DcR3.Fc and HBD.Fc

**Figure 5:**
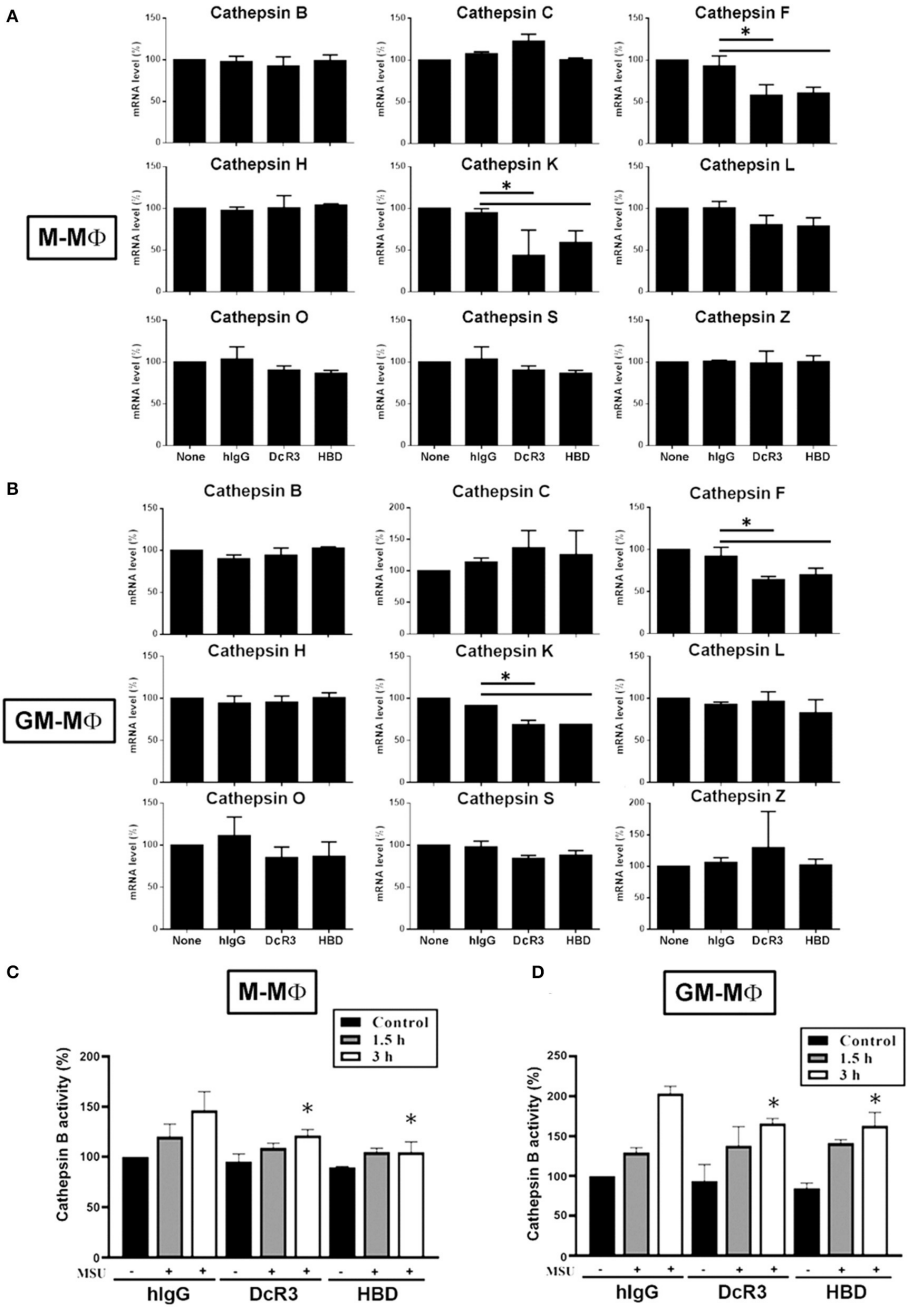
Expression of cysteine cathepsin F and K and activity of cathepsin B were suppressed in DcR3.Fc- or HBD.Fc-treated M-Mϕ and GM-Mϕ. hIgG-, DcR3.Fc-, or HBD.Fc (3 μg/ml for each)-treated 6 day-cultured M-Mϕ **(A,C)** and GM-Mϕ **(B,D)** were harvested, and the total mRNA was extracted. Then, the levels of indicated cysteine cathepsins were analyzed through real-time PCR using specific primers **(A,B)**. Cathepsin B activity was determined in M-Mϕ **(C)** and GM-Mϕ **(D)** after treatment with MSU (300 μg/ml) for 1.5 or 3 h. The data shown were mean ± SD of three independent experiments. The statistical significance was determined by one-way ANOVA. The statistical significance *p < 0.05 was obtained by comparing DcR3.Fc- or HBD.Fc-treated group to hIgG-group.

It has been shown that ruptured lysosomes release cysteine cathepsins to activate NLRP3 inflammasome (21, 22). To examine whether DcR3.Fc and HBD.Fc might alter the release of cysteine cathepsins in MSU-treated macrophages, we analyzed all the cysteine cathepsin isoforms by real-time reverse-transcriptase PCR. Among nine cysteine cathepsins, the transcriptional levels of cathepsin F and K were significantly reduced in DcR3.Fc- or HBD.Fc-treated M-Mϕ and GM-Mϕ, while the expression levels of the other members (cathepsin B, C, H, L, O, S, and Z) were not altered by DcR3.Fc or HBD.Fc (Figures 5A,B). Similar observations were found in the DcR3.Fc- or the HBD.Fc-treated THP-1 macrophages (Supplementary Figure 2A). In addition, because the activation of cathepsin B has been implicated in the NLRP3 inflammasome activation (21, 22), we further determined the activity. We found that MSU-induced cathepsin B activity was inhibited by DcR3.Fc and HBD.Fc in M-Mϕ (Figure 5C) and GM-Mϕ (Figure 5D). Likewise, MSU-induced cathepsin B activation in L929-Mϕ was attenuated by DcR3.Fc (Supplementary Figure 2B). All these observations indicate that DcR3.Fc and HBD.Fc are able to suppress the expression of cathepsins F and K and maintain the integrity of the lysosomal membrane to suppress the release of cathepsins B, thereby inhibiting the MSU-induced NLRP3 inflammasome activation.

### The Suppression of MSU-Induced Mitochondrial Production of the Reactive Oxygen Species by DcR3.Fc and HBD.Fc

**Figure 6:**
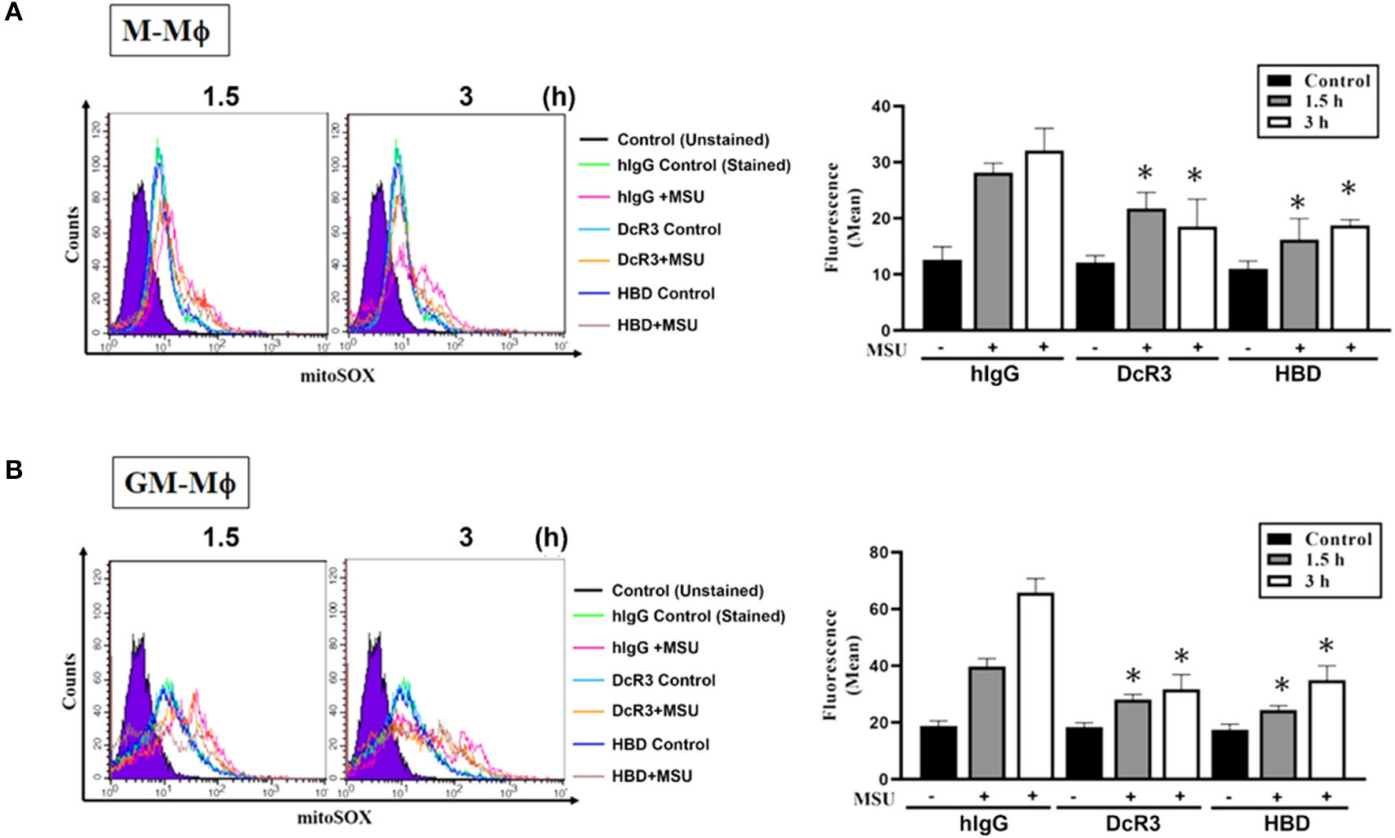
Suppression of MSU-induced mitochondrial production of the ROS by DcR3.Fc and HBD.Fc in M-Mϕ and GM-Mϕ. M-Mϕ **(A)** and GM-Mϕ **(B)** treated with hIgG, DcR3.Fc, or HBD.Fc- (3 μg/ml of each) were stained with MitoSOX (2.5 μM, 30 min) and then stimulated with MSU (300 μg/ml) for 1.5 or 3 h. The MitoSOX fluorescence was detected through the PE-channel by flow cytometry, and the median fluorescence intensity (MFIs) were shown in each histogram. The data shown were mean ± SD of three independent experiments. The statistical significance was determined by one-way ANOVA. *p < 0.05 was obtained by comparing DcR3.Fc- or HBD.Fc-treated group to hIgG-group.

Previous studies showed that MSU can trigger mitochondrial production of the ROS to participate in the activation of the inflammasome. To understand the effects of DcR3.Fc and HBD.Fc on the MSU-induced mitochondrial production of the ROS, the macrophages were stained with MitoSox Red and analyzed by flow cytometry. We found while mitochondrial production of the ROS rapidly increased at 1.5 and 3 h after treatment with MSU (300 μg/ml) in M-Mϕ (Figure 6A) and GM-Mϕ (Figure 6B), it was significantly reduced by DcR3.Fc and HBD.Fc. Similarly, DcR3.Fc and HBD.Fc also showed inhibition in THP-1 (Supplementary Figure 3A) and L929-Mϕ (Supplementary Figure 3B). These results indicate that DcR3.Fc and HBD.Fc not only inhibit MSU-triggered lysosomal rupture but also suppress MSU-induced mitochondrial production of the ROS.

### Less MSU-Induced Inflammatory Cytokines and Immune Cell Infiltration in DcR3-tg Mice

**Figure 7:**
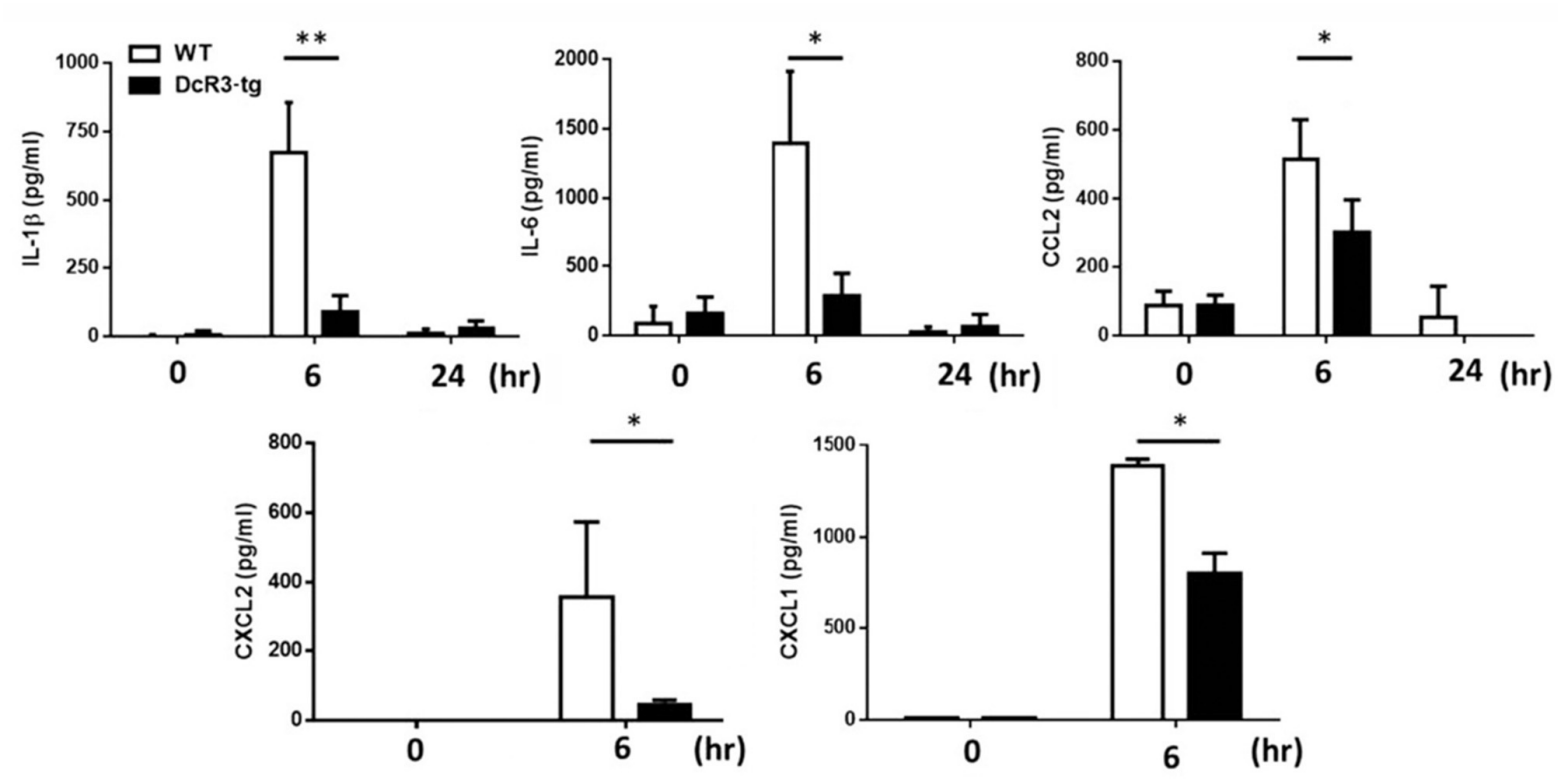
Less amounts of inflammatory cytokines and chemokines were induced in DcR3-tg mice by MSU. MSU (3 mg/mouse) or PBS was injected into the air pouch of DcR3-tg or WT B6 mice. Mice were sacrificed, and the lavage fluid was collected at indicated times. The data shown were mean ± SD of three independent experiments. The statistical significance was determined by the student *t*-test. *p < 0.05 and **p < 0.01 were obtained by comparing DcR3-tg mice to WT mice.

**Figure 8:**
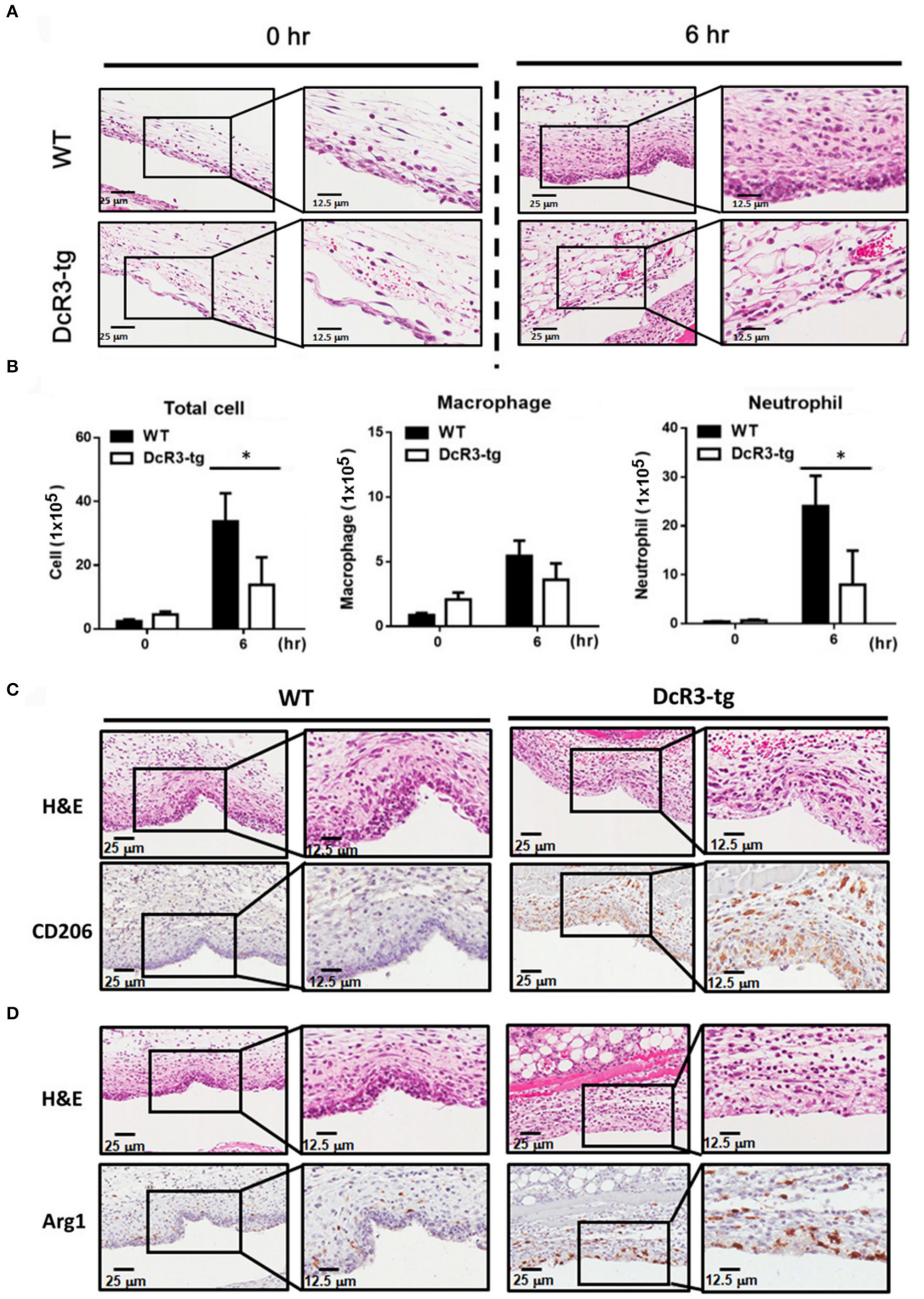
Neutrophil recruitment was reduced, and M2 macrophage was higher in DcR3-tg mice stimulated with MSU. MSU (3 mg/mouse) or PBS only was injected into the air pouch of DcR3-tg or WT mice. After 6 h, the lavage fluid and skin area of air pouch were collected. The skin samples were stained with H&E **(A)**, CD206 **(C)**, or Arg1 **(D)**. The H&E data shown were representative for counter staining. The cells in the lavage fluid were stained with anti-Ly6G (as neutrophil marker) and F4/80 (as macrophage marker) **(B)**. The data shown were mean ± SD of three independent experiments. The statistical significance was determined by the student *t*-test. *p < 0.05 was obtained by comparing DcR3-tg mice to WT mice.

Because IL-1β is a potent proinflammatory cytokine that causes inflammation at the initiation step of gout and neutrophil recruitment, we further asked whether DcR3 can inhibit the MSU crystal-induced inflammation *in vivo*. To address this question, we set up an air pouch model and measured the amount of MSU-induced cytokines and chemokines in WT and DcR3-tg mice. Previously, we have demonstrated the enhanced tumor promotion (13) and decreased Th1 immune response (3) in DcR3-Tg mice where human DcR3 is expressed in mouse myeloid cells and can be released and detected in mouse serum. We found that the levels of proinflammatory cytokines (IL-1β and IL-6) and chemokines (CCL2, CXCL1, and CXCL2) were increased in the air pouch fluid 6 h after the inoculation with MSU. Compared to the WT mice, DcR3-tg mice produced lower amounts of proinflammatory cytokines and chemokines in the lavage fluid (Figure 7). It has been reported that the recruitment of neutrophils is dependent on IL-1β and chemokines (40, 41); thus, we compared cell infiltration in the air pouch membrane between the WT and DcR3-tg mice. We found that abundant cell infiltration into the air pouch membrane was observed in the WT mice at 6 h after inoculation with MSU. However, cell infiltration was significantly decreased in DcR3-tg mice (Figure 8A). While both macrophages (F4/80^+^) and neutrophils (Ly6G^+^) infiltrated into the air pouch membrane after injection of MSU in WT mice, the infiltration of neutrophils constituted about 75% of infiltrated cells in WT mice. We found that the infiltrated neutrophils were attenuated in DcR3-tg mice when compared to WT mice (Figure 8B).

**Figure 9:**
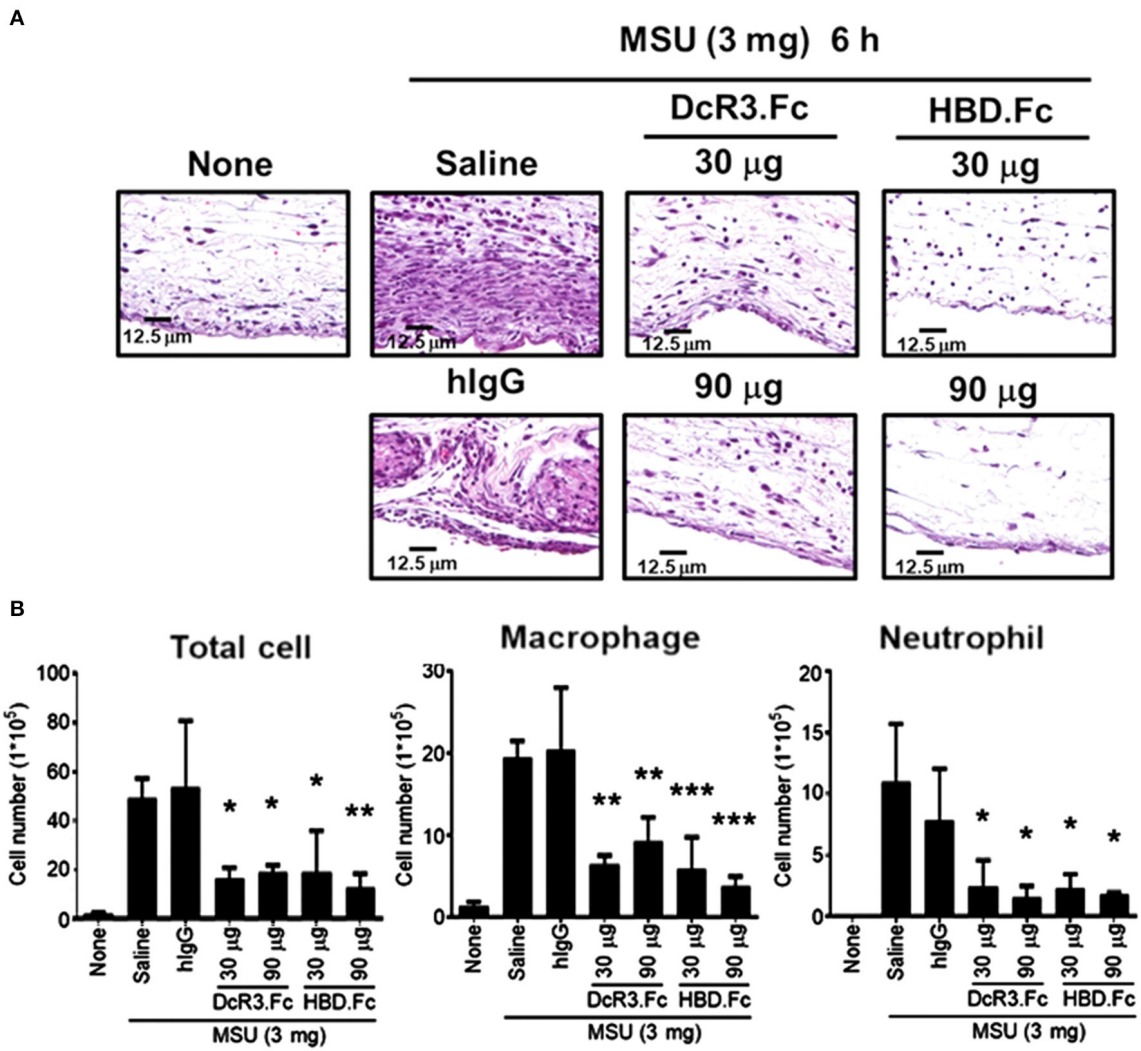
DcR3.Fc and HBD.Fc treatment reduced MSU-induced recruitment of infiltrating immune cells. DcR3.Fc or hIgG1 was injected intravenously into WT mice. After 24 h, MSU (3 mg/mouse) or PBS was injected into the air pouch of WT mice. Mice were sacrificed, and the lavage fluid or the skin area of air pouch was collected 6 h after injection with MSU. The skin samples were stained with H&E **(A)**. The H&E data shown were representative from three independent experiments. The cells in the lavage fluid were counted and stained with anti-Ly6G (as neutrophil marker) and F4/80 (as macrophage marker) **(B)**. The data shown were mean ± SD of three independent experiments. The statistical significance was determined by one-way ANOVA. *p < 0.05, **p < 0.01, and ***p < 0.001 were obtained by comparing DcR3.Fc- or HBD.Fc-treated group to hIgG-group.

We have shown that DcR3.Fc can induce the differentiation of the macrophages into the M2-like phenotype (8, 9), and inflammasome activation is suppressed in the M2 macrophages (42). Thus, we asked whether the MSU-induced infiltrating macrophages are skewered to the M2 phenotype in DcR3-tg mice. As shown in Figures 8C,D, most of the infiltrating cells in WT mice were CD206^−^ and Arg1^−^, while the number of CD206^+^ and Arg1^+^ cells were increased in DcR3-tg mice. To confirm these observations in DcR3.Tg mice, we injected WT mice with hIgG (90 μg), DcR3.Fc (30 or 90 μg), or HBD.Fc (30 or 90 μg) at 24 h before inoculation with MSU (3 mg). We found that the infiltrations of neutrophils and the macrophages were significantly reduced by DcR3.Fc or HBD.Fc (Figures 9A,B). Thus, we conclude that DcR3 has a potent effect to suppress MSU-induced inflammation *in vivo*, and DcR3.Fc and HBD.Fc are promising therapeutic agents to treat diseases caused by particle-induced lysosomal rupture and inflammatory reactions.

## Discussion

In this study, we found that DcR3.Fc and HBD.Fc can attenuate the secretion of IL-1β and NLRP3 inflammasome activation in the macrophages after the stimulation of MSU *in vitro*, and less proinflammatory cytokines and cell infiltration were found in MSU-inoculated DcR3-transgenic mice in the air pouch model. These observations indicate that DcR3.Fc and HBD.Fc can inhibit MSU-induced NLRP3 inflammasome activation to alleviate gout inflammation *via* decoy-independent pathway. We have shown that DcR3 is able to induce M2-like macrophages (6, 8, 9), and DcR3.Fc and HBD.Fc skewed the infiltrating cells into CD206^+^ and Arg1^+^ cells, indicating that DcR3.Fc- and HBD.Fc-mediated effects are also *via* the induction of M2-like macrophages. This observation is in accordance with the fact that the activation of the inflammasome is suppressed in M2 macrophages (42–44). Unlike DcR3.Fc, HBD.Fc is able to bind HSPGs but not three members of DcR3 ligands (FasL, LIGHT, and TL1A) (11). Thus, the DcR3.Fc- and HBD.Fc-mediated immunomodulatory effects are *via* activating HSPGs rather than neutralizing endogenous FasL, LIGHT, and TL1A.

Processing of IL-1β by NLRP3 inflammasome relies on the induction of the gene expressions of pro-IL-1β and NLRP3 and caspase-1 activation by assembling NLRP3 inflammasome components. In this study, we found that DcR3.Fc and HBD.Fc inhibit caspase-1 activation and mitochondrial production of the ROS as well as stabilize lysosomes, rather than the gene expression of NLRP3 or pro-IL-1β. Moreover, the uptake of MSU crystals is not altered by DcR3.Fc or HBD.Fc, suggesting that DcR3.Fc- and HBD.Fc-mediated effect is *via* the assembly of the modulating inflammasome. Moreover, DcR3.Fc and HBD.Fc inhibit mitochondrial production of the ROS and the lysosomal rupture induced by MSU. In contrast, DcR3 is ineffective to affect the activation of the P2X7-mediated inflammasome even though P2X7 activation also leads to the elevation of mitochondrial ROS and rupture of lysosomes (36). This differential effect of DcR3 implies the multifaceted regulatory pathway upstream ROS and lysosome for NLRP3 inflammasome activation and the specific mechanisms underlying DcR3-mediated intervention in crystal-induced cellular events for NLRP3 activation. Therefore, DcR3.Fc and HBD.Fc are potent suppressors in the particle-induced NLRP3 inflammasome activation and IL-1β secretion. It would be very interesting to further ask how DcR3.Fc and HBD.Fc increase lysosomal stability and whether DcR3.Fc and HBD.Fc may be applied to treat particle-associated inflammatory reactions, such as gout and silicosis in the future. Because the size range of MSU and silica is in micrometer (1~10 μm), which is in the same range as atmospheric particles (such as PM_10_ or PM_2.5_) in polluted air, it would be very interesting to ask whether DcR3.Fc and HBD.Fc are also effective to suppress pulmonary inflammation caused by PM_10_ or PM_2.5_.

It has been reported that IL-4 and IL-13-polarized M2 macrophages express less NLRP3 after stimulation of the LPS (44). In addition, ATP-induced inflammasome activation is suppressed in IL-4-polarized M2 macrophages *via* modulating the subcellular localization of NLRP3 and microtubule polymerization (45). Although DcR3.Fc and HBD.Fc induce M2-like macrophages possibly *via* transcriptional mechanism (8, 9), they do not inhibit the expression of LPS-induced NLRP3 and activation of ATP-induced NLRP3 inflammasome, indicating that DcR3.Fc- and HBD.Fc-mediated signaling and action mode are different from those of IL-4. Moreover, the anti-inflammatory immune modulation action of DcR3 was evident in the gout model in MSU-elicited mice. DcR3-transgenic mice produce less amounts of IL-1β, IL-6, CXCL1, CXCL2, and CCL2 and display less neutrophil infiltration in an air pouch model after inoculation with MSU than WT mice. Although the total number of increased macrophage infiltration in the air pouch model in mice was not changed by DcR3, we unexpectedly found higher M2 phenotype cells with CD206 and Arg1 positive staining in the air pouch area of DcR3 group. This suggests that M1/M2 polarization shift might exist within 24 h after injection with MSU in this air pouch model. Unlike other inflammatory disease models, MSU-induced inflammation in the air pouch model displays rapid onset and recovery. Pessler et al. found that the number of infiltrated leukocytes in the air pouch membrane rose from 0 to 9 h after injection of MSU and declined almost to the resting state at 50 h (37). Because of this cellular immune feature, it is difficult to analyze the immune responses after 24 h. So, we currently do not have further mechanistic explanations for the M1/M2 shift regulated by DcR3 in the air pouch model. Nevertheless, the ability of DcR3 to upregulate IL-4 and IL-10 but simultaneously downregulate IFN-γ, IL-12, TNFα, and IL-17 after influenza hemagglutinin peptide stimulation (3) and in the experimental autoimmune encephalomyelitis model (14) was demonstrated. Cellular studies also revealed that DcR3 can increase production of IL-4 from T cells (46). All these findings suggest that DcR3 can reciprocally regulate M1 and M2 cytokines and shift macrophages toward M2 polarization.

Lysosomal cysteine cathepsins are a family of proteases that are activated after proteolytic degradation in lysosomes. Among the cathepsin family, cathepsin B contributes to the activation of NLRP3 inflammasome (21, 22, 47). Moreover, recent studies show that pan cysteine cathepsin inhibitors can suppress crystal-induced IL-1β secretion in cathepsin B-deficient macrophages (21), suggesting other cysteine cathepsins also participate in crystal-induced inflammasome activation. Moreover, the release of cathepsin C, L, S, and Z from the destabilized lysosomes have been implicated in the activation of inflammasome (22, 38, 48, 49). In this study, we found that the cathepsin B activity, but not the expression level of cathepsin B, is downregulated by DcR3.Fc. Moreover, the expressions of cysteine cathepsins F and K were also downregulated by DcR3 in macrophages. Thus, DcR3.Fc and HBD.Fc are able to inhibit the activation of the inflammasome *via* maintaining lysosomal integrity and modulating the expression and activity of the cathepsin family.

Taken together, both endogenous DcR3 and exogenous DcR3.Fc and HBD.Fc are able to inhibit the activation of the MSU-induced inflammasome *in vitro* and *in vivo*. DcR3.Fc and HBD.Fc are able to inhibit mitochondrial production of the ROS, stabilize lysosome integrity, attenuate the activity of cathepsins, and downregulate the activity of caspase-1 without affecting the crystal uptake and expression of NLRP3/pro-IL-1β. Thus, recombinant DcR3.Fc and HBD.Fc have great potential to become therapeutic agents to treat microparticle-induced inflammatory diseases in future.

## Data Availability Statement

The raw data supporting the conclusions of this article will be made available by the authors, without undue reservation.

## Ethics Statement

The animal study was reviewed and approved by Mice experiments were conducted in accordance with institute regulation after receiving approval from the Ethics Committee of the National Taiwan University College of Medicine (No. 20180091).

## Author Contributions

Y-GP, W-WL, and S-LH: conceptualization, validation, writing, review, and editing. Y-GP, M-TH, PS, and D-YH: methodology. Y-GP and PS: software. Y-GP, M-TH, PS, D-YH, W-WL, and S-LH: formal analysis. W-WL and S-LH: supervision and funding acquisition. All authors have read and agreed to the published version of the manuscript.

## Conflict of Interest

The authors declare that the research was conducted in the absence of any commercial or financial relationships that could be construed as a potential conflict of interest.

## Supplementary Material

Supplementary Figure 1 DcR3.Fc and HBD.Fc inhibited MSU-induced IL-1 production in THP-1 and U937 cells. THP-1 and U937 cells were differentiated with PMA (3 nM) under treatment with hIgG, DcR3.Fc, or HBD.Fc for 24 h. In THP-1 cells, two concentrations (1 or 3 μg/ml) of DcR3.Fc or HBD.Fc were used, while in U937 cells 3 μg/ml was used in U937 cells. In both cell types, the concentration of hIgG was 3 μg/ml. The adherent cells were primed with LPS (100 ng/ml) for 4 h and then stimulation with MSU (150 or 300 μg/ml) for 4 h. The concentration of IL-1β was measured by ELISA. All data shown were mean ± SD from three independent experiments. The statistical significance was determined by one-way ANOVA. ^*^*p* < 0.05 and ^**^*p* < 0.01 were obtained by comparing DcR3.Fc- or HBD.Fc-treated group to hIgG-group. “NS” means no statistical significance.

Supplementary Figure 2 Effects of DcR3.Fc and HBD.Fc on the expression of cathepsins and the activity in THP-1 and L929-Mϕ. **(A)** THP-1 cells were differentiated with PMA (3 nM) under treatment with hIgG, DcR3.Fc, or HBD.Fc (3 μg/ml for each) treatment for 24 h. The gene expressions of cathepsin B, F, and K were determined. **(B)** L929-Mϕ after treatment with hIgG or DcR3.Fc (3 μg/ml) were stimulated with MSU (300 μg/ml) for 3 h for cathepsin B activity assay. The data shown were mean ± SD of three independent experiments. The statistical significance was determined by one-way ANOVA. ^*^*p* < 0.05 and ^**^*p* < 0.01 were obtained by comparing DcR3.Fc- or HBD.Fc-treated group to hIgG-group.

Supplementary Figure 3 Suppression of MSU-induced mitochondrial production of the ROS by DcR3.Fc and HBD.Fc in THP-1 and L929-Mϕ. **(A)** hIgG- (3 μg/ml), DcR3.Fc- (3 μg/ml), or HBD.Fc- (3 μg/ml) treated PMA-differentiated THP-1 cells were stained with MitoSOX (2.5 μM, 30 min) and then treated with MSU (300 μg/ml) for indicated times. **(B)** A similar experiment was conducted in L929-Mϕ. The MitoSOX fluorescence was detected through the PE-channel by flow cytometry, and the MFIs were shown in each histogram. All data shown were representative from three independent experiments. The data shown were mean ± SD of three independent experiments. The statistical significance was determined by one-way ANOVA. ^*^*p* < 0.05 was obtained by comparing DcR3.Fc- or HBD.Fc-treated group to hIgG-group.
