# Supplementary figures and images for: Decoy Receptor 3 Inhibits Monosodium Urate-Induced NLRP3 Inflammasome Activation via Reduction of Reactive Oxygen Species Production and Lysosomal Rupture
Source: Front Immunol. 2021 Mar 3;12:638676. doi: 10.3389/fimmu.2021.638676 (PMC7966727; doi:10.3389/fimmu.2021.638676)

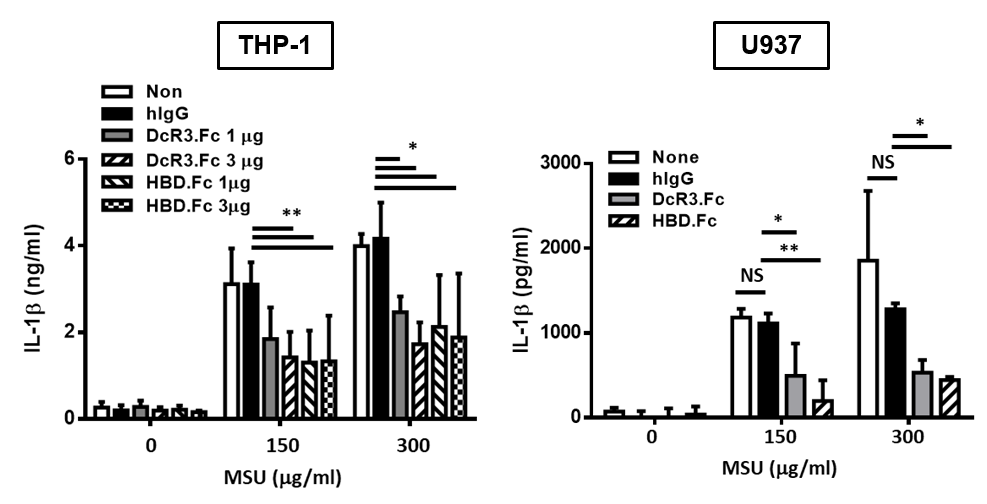

Supplement: Supplementary Figure 1 — DcR3.Fc and HBD.Fc inhibited MSU-induced IL-1 production in THP-1 and U937 cells. THP-1 and U937 cells were differentiated with PMA (3 nM) under treatment with hIgG, DcR3.Fc, or HBD.Fc for 24 h. In THP-1 cells, two concentrations (1 or 3 μg/ml) of DcR3.Fc or HBD.Fc were used, while in U937 cells 3 μg/ml was used in U937 cells. In both cell types, the concentration of hIgG was 3 μg/ml. The adherent cells were primed with LPS (100 ng/ml) for 4 h and then stimulation with MSU (150 or 300 μg/ml) for 4 h. The concentration of IL-1β was measured by ELISA. All data shown were mean ± SD from three independent experiments. The statistical significance was determined by one-way ANOVA. *p < 0.05 and **p < 0.01 were obtained by comparing DcR3.Fc- or HBD.Fc-treated group to hIgG-group. “NS” means no statistical significance. [file Image_1.TIF]

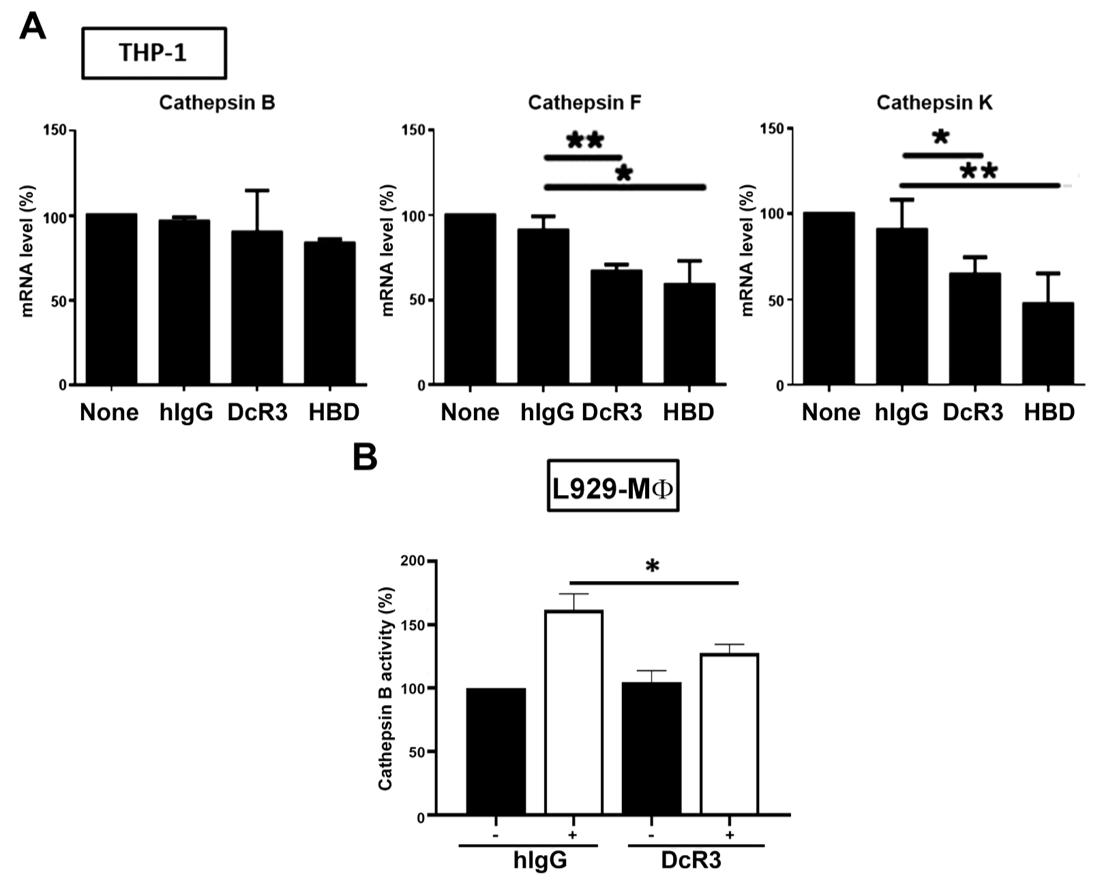

Supplement: Supplementary Figure 2 — Effects of DcR3.Fc and HBD.Fc on the expression of cathepsins and the activity in THP-1 and L929-Mϕ. (A) THP-1 cells were differentiated with PMA (3 nM) under treatment with hIgG, DcR3.Fc, or HBD.Fc (3 μg/ml for each) treatment for 24 h. The gene expressions of cathepsin B, F, and K were determined. (B) L929-Mϕ after treatment with hIgG or DcR3.Fc (3 μg/ml) were stimulated with MSU (300 μg/ml) for 3 h for cathepsin B activity assay. The data shown were mean ± SD of three independent experiments. The statistical significance was determined by one-way ANOVA. *p < 0.05 and **p < 0.01 were obtained by comparing DcR3.Fc- or HBD.Fc-treated group to hIgG-group. [file Image_2.TIF]

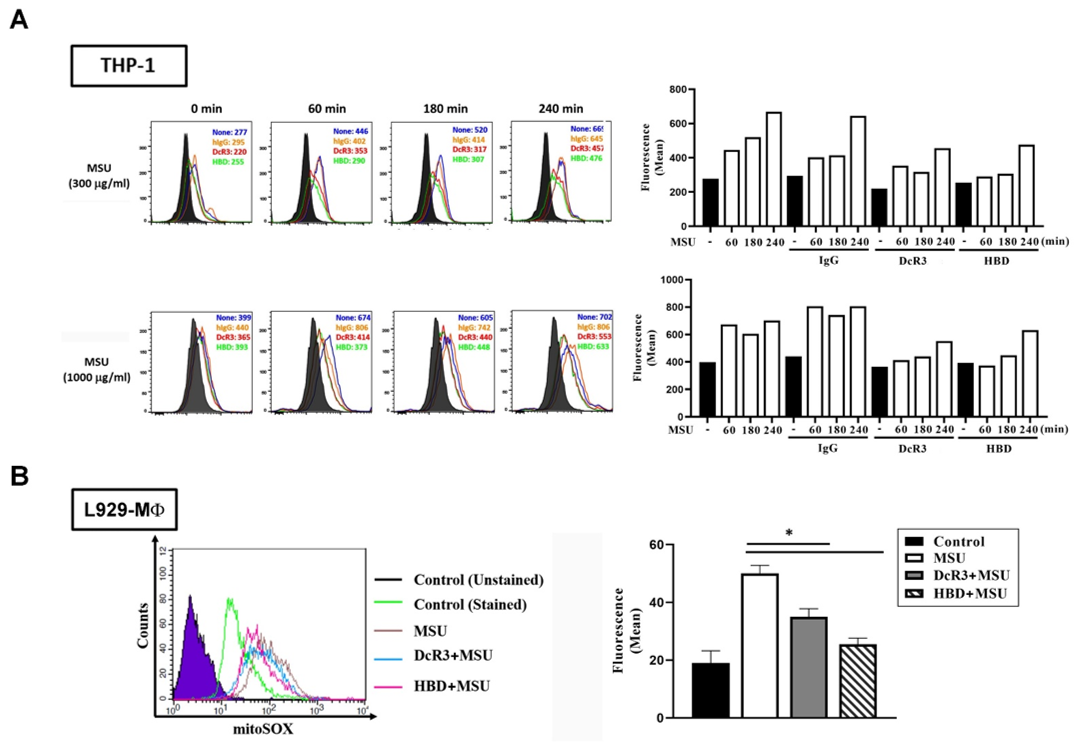

Supplement: Supplementary Figure 3 — Suppression of MSU-induced mitochondrial production of the ROS by DcR3.Fc and HBD.Fc in THP-1 and L929-Mϕ. (A) hIgG- (3 μg/ml), DcR3.Fc- (3 μg/ml), or HBD.Fc- (3 μg/ml) treated PMA-differentiated THP-1 cells were stained with MitoSOX (2.5 μM, 30 min) and then treated with MSU (300 μg/ml) for indicated times. (B) A similar experiment was conducted in L929-Mϕ. The MitoSOX fluorescence was detected through the PE-channel by flow cytometry, and the MFIs were shown in each histogram. All data shown were representative from three independent experiments. The data shown were mean ± SD of three independent experiments. The statistical significance was determined by one-way ANOVA. *p < 0.05 was obtained by comparing DcR3.Fc- or HBD.Fc-treated group to hIgG-group. [file Image_3.TIF]
